# The effects of creative drama activities on preschoolers' peer relationships and humor skills and the predictive role of humor

**DOI:** 10.3389/fpsyg.2026.1892073

**Published:** 2026-07-20

**Authors:** Özlem Bal

**Affiliations:** Sungurlu Vocational School, Hitit University, Çorum, Türkiye

**Keywords:** child development, creative drama, humor, peer relationships, preschool

## Abstract

**Introduction:**

Peer relationships and humor skills may be important for the social-emotional skills of preschool-aged children. Creative drama activities offer opportunities for role-playing, social interaction, and self-expression through imagination, which can support the development of these skills. However, studies examining peer relationships and humor skills together within a creative drama framework remain limited. This study aimed to investigate the effects of creative drama activities on preschool children's peer relationships and humor skills and to examine the predictive role of humor skills on peer relationships.

**Methods:**

A mixed-methods design was employed. The study group consisted of 17 children in the experimental group and 21 children in the control group. Children in the experimental group participated in 16 creative drama sessions conducted over an eight-week period. Quantitative data were collected using the Preschool Peer Relationships Scale and the Humor Scale, while qualitative data were obtained through parent interviews, teacher interviews, and field notes.

**Results:**

The research findings revealed that children in the experimental group showed greater improvements in humor skills and social interaction a subdimension of peer relationships compared to those in the control group. These improvements were largely maintained during the follow-up phase. Significant positive correlations were found between humor skills and the social interaction dimension of peer relationships. Qualitative findings supported the quantitative results by indicating improvements in some social skills, and use of humor.

**Discussion:**

Overall, the findings suggest that creative drama activities may be an effective approach for supporting preschool children's social interaction and humor skills. The study also highlights the potential role of humor skills in fostering positive peer interactions during early childhood.

## 1 Introduction

The preschool period represents an important stage in children's development during which social, emotional, cognitive, and behavioral competencies continue to expand and become more sophisticated. Experiences during this period contribute substantially to children's emerging social adaptation, communication patterns, and interpersonal relationships. Preschool education settings, in particular, provide valuable opportunities for children to interact with peers, assume social roles, and develop social skills through active participation and shared experiences (Howard and Melhuish, 2017; Atiş Akyol and Aşkar, 2022).

It is indicated that peer relations are defined as social interactions involving reciprocity and continuity between children of similar age and developmental level and are one of the fundamental determinants of social development in early childhood (Gülay Ogelman and Sari, 2024). The children are learning basic social-emotional skills such as sharing, cooperation, empathy, understanding and regulating emotions, and conflict resolution through their relationships established with the peers (Pepler and Bierman, 2018; Taylor et al., 2023). It is argued that quality peer relationships increase children's social acknowledgment, facilitate school adaptation and support their psychological well-being; on the other hand, negative experiences such as peer rejection and exclusion may pose long-term risks to children's social and emotional development (Cillessen and Bellmore, 2022; Lariviere-Bastien et al., 2022; Lorijn et al., 2022).

The quality of peer relationships is closely associated with children's social positions within the group (Gülay Ogelman and Sari, 2024).

As a matter of fact, it is pointed out that children who are accepted by their peers, tend to be cooperative, adaptable, empathetic, and have a well-developed sense of humor (Ladd, 2009). In this context, humor is considered as an important interaction tool that supports the social acknowledgment of children within the peer group. Studies conducted suggest that there are positive relationships between the use of humor and social skills, empathy and interpersonal communication (Wu et al., 2021; Semrud-Clikeman and Glass, 2010).

Humor is defined not merely as an emotional state dependent on entertainment, but as a multifaceted social competence encompassing an individual's ability to recognize, produce, and share what is funny with others (Bag, 2020). It is pointed out that humor in early childhood has functions such as initiating and maintaining peer interactions, strengthening social ties and supporting intra-group harmony (Loizou and Loizou, 2019; Ylikörkkö et al., 2026). From this aspect, humor is considered as a skill that is actively used in children's social interaction processes and develops through social learning (Paine et al., 2025).

At this point, drama-based interventions stand out as one of the structured learning environments that support children's social interactions. Drama is defined as the interpretation and animation of a word, concept, behavior or experience through theatrical techniques and playful processes (San, 1990). Drama in early childhood education comes forward as an important pedagogical approach that supports children's active participation and makes the learning process experiential. Creative drama engages children in active social interaction, role-taking, and collaborative problem-solving processes, which may support the development of social understanding, emotional expression, and interpersonal competence during the preschool years (Adigüzel, 2017; McCaslin, 2016).

Drama activities enrich children's social interactions by improving their skills in role-playing, self-expression, and understanding others' perspectives. In this process, children experience social skills such as cooperating with their peers, initiating and maintaining communication in a natural learning environment. In addition, drama-based activities are believed to have an important potential in terms of supporting children's creative thinking processes and preparing the ground for humor generation (Martin and Ford, 2018; Colliver and Veraksa, 2021; Sanchez et al., 2026; Paine et al., 2025).

From a sociocultural theory perspective, creative drama is a social learning environment that enables children to collaborate with their peers, take on different roles, and construct shared meanings. In this process, children evaluate others' perspectives, interpret social situations, and use their communication skills (Celume et al., 2020). Similarly, it has been noted that the development of humor is linked to processes such as social cognition, perspective-taking, imagination, and flexible thinking (Bergen, 2019; Hoicka and Akhtar, 2012). Therefore, creative drama can activate shared developmental processes that support the development of both humor skills and positive peer relationships.

Previous research has shown that drama-based interventions support children's social-emotional skills, such as social understanding, social problem-solving, collaboration, self-regulation, and peer relationships (Celume et al., 2020; Robertson et al., 2020; Ozen-Uyar et al., 2025; Boal-Palheiros and Ilari, 2023; Akalin and Boz, 2024). However, studies examining the effect of creative drama on humor skills are quite limited. Furthermore, research that simultaneously examines the relationships among creative drama, humor skills, and peer relationships in the preschool period is rarely found. This situation necessitates a joint examination of the effects of creative drama on both humor skills and peer relationships.

In this context, this study examines the effect of drama activities on peer relations and humor skills in preschool children, and also discusses the predictor relationships between peer relations and humor skills.

## 2 Peer relations humor and drama

Peer relationships in early childhood play a central part in helping children make sense of the social world and develop their interpersonal skills. These relationships not only offer social interaction opportunities, but also contribute to the development of children's skills in regulating their behavior, expressing their emotions, and understanding others' perspectives (Gülay Ogelman and Sari, 2024; Pepler and Bierman, 2018).

The quality of peer interactions is an important factor determining children's social positions in the group and is decisive in the formation of social roles such as acceptance, exclusion or leadership (Cillessen and Bellmore, 2022). In this connection, fostering peer relationships in early childhood is critical for both existing and future social adaptation of children; in this process, plays and structured interaction environments offer important learning spaces in which children develop their social skills and experience social roles (Lariviere-Bastien et al., 2022; Prins et al., 2022). On the other hand, negative experiences in peer relationships can pose a risk to children's development. Peer rejection, exclusion or negative social interactions can weaken children's self-perception, negatively impact their social adaptation and increase their feelings of loneliness (Yavuzer, 2016). Studies reveal that introverted children who are not accepted by their peers and therefore feel lonely are more likely to experience emotional problems such as depression and anxiety, while children who exhibit aggressive behavior toward their peers are at a higher risk of showing problematic actions such as dropping out of school and engaging in delinquent activities (Santrock, 2015).

When considered within the context of peer relationships, humor may serve as an important means of social interaction and communication among children. Humorous exchanges can provide opportunities for shared enjoyment, support social engagement, and contribute to positive peer experiences (Semrud-Clikeman and Glass, 2010; Yunus and Dalli, 2019; Paine et al., 2025).

Specifically, behaviors such as laughing together, joking with one another, and creating humorous elements in a play contribute to the development of closeness and a sense of belonging among children (Ylikörkkö et al., 2026). Play and humor are considered two processes that are closely related from a developmental perspective. According to Hammershøj (2021), both processes involve the creation of alternative meanings, the reinterpretation of everyday experiences, and the discovery of unexpected situations. Therefore, while play enables children to utilize their creativity, imagination, and different perspectives, humor similarly relies on their ability to recognize incongruities and generate new meanings. Loizou (*2025*) notes that children's production of humor is largely related to their ability to recognize and create incongruities. Creative drama activities including role-playing, improvisation, symbolic transformation, and imaginary scenarios can provide children with opportunities to experience such situations, thereby creating a suitable environment for the development of humor skills (Hammershøj, 2021; Loizou, 2025).

It is argued that understanding and creating humor in childhood is strongly linked to cognitive processes and areas of social development (Hoicka and Akhtar, 2012). In order to effectively benefit from the social and emotional functions of humor, children must have achieved a certain level of empathy, emotion regulation, and interpersonal problem-solving skills (Mireault and Reddy, 2016). In the case study conducted by Loizou and Loizou (2019), it was put forward that humor-based structured activities applied to children between the ages of 3.5 and 4.5 increase both the amount and originality of children's humorous creations and support their creative thinking processes. As a result of the study carried out by Rönkkö and Aerila (2018), it was found that individual and collaborative humor-based holistic art activities contributed to supporting preschool children's learning processes, revealing their creativity, generating ideas and interacting with their peers. One of the effective approaches that support two important social processes in early childhood education, such as humor and peer relationships, is drama-based interventions.

Drama activities provide children with the opportunity to communicate, cooperate and take on social roles with their peers by offering a structured yet flexible interaction environment (San, 1990; Adigüzel, 2017). Through techniques such as role-playing and improvisation, children experience different perspectives and develop awareness of social situations. In this process, both verbal and non-verbal communication skills of children are supported while their social interactions become more meaningful and sustainable (McCaslin, 2016). Another potential contribution of drama activities is that they encourage imagination, creative thinking, and flexible responses through role-taking and perspective-shifting experiences (Adigüzel, 2017; McCaslin, 2016). These skills have been associated with humor development, as understanding and producing humor often require the recognition of incongruity, alternative perspectives, and flexible interpretation of situations (Bergen, 2019; Semrud-Clikeman and Glass, 2010). The ability to react differently and creatively to the unexpected situations, to approach events from different perspectives, and to think symbolically are common in both drama processes and humor creation (Martin and Ford, 2018). Therefore, drama environments are considered to provide a natural and supportive context for children to develop their humor skills. Indeed, the interactions that emerge naturally during the drama process allow children to create humor and share this humor with their peers (Paine et al., 2025).

Creative drama is an effective method that holistically supports cognitive, sensory and social development (Liu, 2023). Through drama, children participate in role-playing, improvisation, and script-based interactions, allowing them to experience real-life situations which help them concretize and internalize abstract concepts (Hu and Shu, 2025). Moreover, creative drama stands out as an efficient teaching method that concentrates on the active participation of the learner, allowing individuals to construct their own knowledge and build new learning upon their existing knowledge. In this respect, it offers a social learning environment while increasing the participation of students in the class (Danckwardt-Lillieström et al., 2020). It is purported that 21st-century skills expected of individuals today, such as creativity, communication, self-expression, problem-solving, critical thinking, and empathy, can be further developed through drama (Atiş Akyol and Aşkar, 2022).

It is understood that children, who are more socially accepted and participate more actively in peer interactions have more opportunities to create humor and participate in humorous interactions; on the other hand, children, who are more socially shy or excluded have less access to such interactions (Ladd, 2009; Paine et al., 2025). Nevertheless, studies examining the simultaneous effects of creative drama-based interventions on these two variables are limited in the literature, while the direction and strength of the relationship between peer relationships and humor skills are not directly addressed in most studies. In this context, examining the interactions between peer relationships, humor, and drama in early childhood through a holistic approach will offer significant contributions, both theoretically and practically. This study intends to reveal the contribution of creative drama-based practices on children's peer relationships and humor skills by addressing these variables together and to account for the predictor relationships between peer relationships and humor skills.

## 3 The present study

The main objective of this study is to investigate the effect of creative drama activities on peer relationships and humor skills of preschool children. Although previous studies have examined the effects of drama-based interventions on children's social competence, collaborative behavior, social understanding, and peer relationships (Celume et al., 2020; Ozen-Uyar et al., 2025), and other studies have investigated the role of humor in children's social interactions and peer experiences (Paine et al., 2021, 2025), research simultaneously examining creative drama, humor skills, and peer relationships in preschool children remains limited. In particular, little is known about how changes in humor skills are associated with changes in children's peer relationships within the context of drama-based interventions.

It is noteworthy that studies investigating the connection of humor with social relations in an experimental framework, especially in the preschool period, are insufficient. Moreover, studies simultaneously examining creative drama, humor skills, and peer relationships in Turkish preschool settings remain limited. In this context, the present study aims to contribute to the literature as a quasi-experimental and mixed method research conducted with pre-school children coming from families at a middle socioeconomic level in Çorum province in Türkiye. A pre-test-post-test-follow-up test design with experimental and control groups was applied in the quantitative phase of the study. In the qualitative phase, parent interviews, teacher interviews, and field notes were used to support and enrich the interpretation of the quantitative findings.

The first objective of the study is to examine the effect of creative drama activities on children's peer relationships and humor skills. Based on the relevant literature, children engaging in drama activities are expected to develop in peer relations dimensions such as sharing, cooperating, developing positive social interaction and peer acceptance. Similarly, it is believed that drama related processes will support children's ability to create humor, understand humor, and interact with humor.

The second objective of the study is to examine the predictor relationships between peer relationships and humor skills. Given that humor is a social tool that enhances social acknowledgment, facilitates communication, and promotes group interaction, children with stronger peer relationships are expected to exhibit improved humor skills. In a similar way, it is thought that humor skills may be a variable that supports positive peer interactions. In this direction, this study examines the degree to which peer relationships and humor skills predict each other. Finally, the present study aims not only to reveal quantitative changes, but also to comprehend more deeply the transformations that occur in children's social interactions and humorous behaviors during the drama process through teacher, family and researcher observations.

## 4 Method

### 4.1 Study population

The study population consists of pre-school children attending the kindergarten of an official primary school affiliated to the Ministry of National Education in Çorum province in the fall semester of the 2025–2026 academic year. Purposive sampling, one of the non-probability-based sampling methods, was applied as part of the study (Sahin, 2014). In order ensure the smooth running of the implementation process and the healthy continuation of the data collection process, an accessible school that can provide implementation integrity was preferred in the study. The experimental group was selected from children attending the morning session, while the control group was selected from children attending the afternoon session in order to prevent the population from influencing each other.

An *a priori* power analysis was conducted using G^*^Power 3.1 (Heinrich Heine University Düsseldorf, Düsseldorf, Germany) to determine the required sample size. The analysis was based on a medium effect size (*d* = 0.50), a significance level of 0.05, and a statistical power of 0.80 (Cohen, 1988). The results indicated that a minimum of 34 participants (17 per group) was required. The final sample consisted of 38 children, including 17 children in the experimental group and 21 children in the control group. The experimental group consisted of 17 children (9 girls and 8 boys) with a mean age of 68.12 months (SD = 1.41), whereas the control group consisted of 21 children (11 girls and 10 boys) with a mean age of 68.14 months (SD = 1.35). The ages of the participants ranged from 66 months to 71 months. Information about the parents was obtained during the interviews. While most of the parents had completed compulsory high school, a smaller proportion held a college degree. Fathers were primarily self-employed, shopkeepers, or civil servants, while most mothers were homemakers. Based on the parents' educational and occupational characteristics, the families participating in the study were considered to be of middle socioeconomic status. The details are shown in Table 1.

**Table 1:** Child and parent information.

Variable	Experimental (*n* = 17)	Control (*n* = 21)
Girls	9	11
Boys	8	10
Mean age (months)	68.12 ± 1.41	68.14 ± 1.35
Father's high school education	13	14
Father's university education	4	7
Mother's high school education	15	16
Mother's university education	2	5
Father civil servant	4	6
Father self-employed	9	12
Father tradesman	3	3
Father unemployed	1	0
Mother homemaker	15	14
Mother teacher	2	2
Mother nurse	0	1
Mother self-employed	0	4

### 4.2 Data collection tools

In this study, the “Preschool Peer Relationship Questionnaire,” “Children's Humor Questionnaire,” “Parent and Teacher Interview Questions,” and “Field Notes” were used.

#### 4.2.1 Preschool peer relationship questionnaire

The Preschool Peer Relationships Scale–Teacher Form was developed to assess peer relationships among preschool children. The scale consists of 37 items rated on a five-point Likert scale ranging from “Never” to “Always.” It includes three subdimensions: bully Child, Friendship and Interaction (Social Interaction), and Victim Child. Sample items include “Displays hurtful behaviors toward peers (e.g., embarrassing, shouting at, or upsetting them)” and “Can initiate communication with peers without adult support.” Higher scores on the Friendship and Interaction subdimension indicate more positive peer relationships, whereas higher scores on the Bully Child and Victim Child subdimensions indicate more problematic peer relationships. The reliability coefficients of the questionnaire were reported as 0.89 for the parent form and 0.97 for the teacher form (Kanmaz and Tezel-Sahin, 2022).

#### 4.2.2 . Children's humor questionnaire

The Children's Humor Questionnaire–Teacher Form, developed to determine preschool children's humor tendencies, is a five-point Likert-type questionnaire consisting of 22 items. The questionnaire includes four sub-dimensions: understanding humor (e.g., identifying humorous situations), preferring humor (e.g., showing interest in humorous content), producing humor (e.g., generating humorous expressions), and benefiting from humor (e.g., using humor in everyday interactions or coping situations). The internal consistency coefficient of the questionnaire was reported as 0.90 (Özyürek et al., 2024).

#### 4.2.3 . Interview questions

Semi-structured interviews were applied to the qualitative aspect of the research. This method allows participants to convey their experiences with their own expressions (Merriam, 2018; Patton, 2014). Parent and teacher interview questions designed by the researcher were regulated in line with the opinions of three experts in the field of pre-school education. The seven questions designed at the initial stage were combined to finalize the interview form, which consisted of three questions. Two of the questions are about peer relationships and one is about humor skills.

#### 4.2.4 Field notes

During the implementation of drama activities, field notes were taken by the researcher. Field notes allow the description of events that occurred during the research process and the re-evaluation of observations made during the analysis phase (Patton, 2014). Short notes and video images taken by the researcher during the observation process were then transcribed (Merriam, 2018). In addition, the responses given to the assessment questions directed to the children after each activity were recorded with voice recording and compiled into notes revealing notable peer relationships and humorous behaviors throughout the day.

### 4.3 Supporting peer relationships and humor skills in preschool children designing creative drama activities

This study examines the effect of drama activities on peer relationships and humor skills, as well as the predictive relationships between humor and peer relationships. It has been noted that social interactions supported by drama enhance the quality of children's peer relationships (Adigüzel, 2017; Atiş Akyol and Aşkar, 2022). According to Bergen (2019), humorous behaviors in children develop largely through play processes and social interactions. In this context, the drama activities designed are structured as organized processes that allow children to interact with their peers and utilize their humorous expression skills. According to Dorothy Heathcote, the key components of drama planning include the theme/learning area, content, roles, framework/outline, materials, and strategies (Adigüzel, 2017). The creative drama process consists of three main stages: “warm-up/preparation,” “enactment,” and “evaluation/discussion” (Adigüzel, 2017; Arveklev et al., 2018). The warm-up/preparation phase aims to help children actively use their senses, make observations, interact with one another, and build trust within the group (Köksal Akyol, 2003; McCaslin, 2016). The enactment phase is the practical process in which children actively participate through improvisation, role-playing, and various drama techniques in line with the chosen theme. The goal of this phase is for children to engage in social interaction, express their feelings and thoughts, experience problem situations, and understand different perspectives (Adigüzel, 2017). In the evaluation phase, the goal is for children to reflect on their experiences during the activity, share their feelings and thoughts, and gain awareness about the process (Köksal Akyol, 2003; Önder, 2010). Drama-based activities offer children the opportunity to get to know both themselves and their peers better. It is noted that drama activities conducted through group work and games support skills such as taking another's perspective, developing empathy, expressing feelings and thoughts, and analyzing social situations (Önder, 2010). In this study, creative drama is approached as an experience-based, child-centered method that supports children's learning through social interaction. The activities were designed to provide experiences where children can naturally develop their peer relationships through play, humor, and group interaction. In this context, the relevant literature was first reviewed, and general objectives aimed at supporting peer relationships and humor skills were established based on the findings. The 16 drama activities developed were submitted for review to three experts in the field, independent of the research; following the necessary revisions, the implementation process was initiated. A summary of the activities is provided in Appendix 1.

### 4.4 Data collection

The present study contributes to the literature by examining the effects of creative drama activities on preschool children's peer relationships and humor skills through a mixed-methods approach. The use of an experimental-control group design with pre-test, post-test, and follow-up measurements enabled the evaluation of both the immediate and sustained effects of the intervention. In addition, quantitative findings were supported through parent interviews, teacher interviews, and field notes, providing a more comprehensive understanding of children's social interaction processes. Another strength of the study is its focus on humor skills, an area that has received relatively limited attention in preschool research, and its examination of the relationship between humor skills and peer relationships.

Despite these strengths, several limitations should be acknowledged. First, the drama activities were developed and implemented by the researcher; therefore, further studies are needed to examine their effectiveness with different practitioners and samples. Although the sample size met the minimum requirement indicated by the power analysis, the relatively small number of participants limits the generalizability of the findings. In addition, the study was conducted only with children from families of middle socioeconomic status in a single district of Türkiye, and the analyses did not consider potential age- or gender-related differences.

Another limitation concerns data collection. Both peer relationships and humor skills were assessed through teacher-reported measures, which may have increased the risk of common method variance and single-source bias. Furthermore, teacher ratings inevitably involve subjective interpretations. Future studies would benefit from incorporating multiple informants, such as parents and teachers, to obtain a more comprehensive assessment of children's behaviors. Qualitative data were collected only from the experimental group, preventing qualitative comparisons between the experimental and control groups. In addition, some parent interviews were limited in duration, which may have restricted the depth of the qualitative data obtained. The participants were assigned according to their existing classroom structures; therefore, full randomization could not be achieved. Moreover, the field notes were recorded by the researcher who conducted the intervention, making it impossible to completely rule out researcher bias in the interpretation of observational data. Due to the nature of the drama-based intervention and the informed consent procedures required when working with young children, a blinding procedure could not be implemented. Consequently, the findings should be interpreted within the context of a natural educational setting. Finally, caution is warranted when generalizing the findings to children from different socioeconomic and cultural backgrounds.

### 4.5 Data analysis

According to this study, which aims to support peer relationships and humor skills of preschool children through drama activities, the findings obtained by quantitative methods were endorsed by qualitative data. SPSS software was used to analyze the quantitative data. Prior to the analyses, normality assumptions were evaluated using skewness and kurtosis coefficients as well as the Shapiro–Wilk test. Values between −1.5 and +1.5 were considered acceptable for skewness and kurtosis. Although some variables demonstrated acceptable skewness and kurtosis values, the Shapiro–Wilk test indicated that a substantial proportion of the variables deviated from a normal distribution (*p* < 0.05). Therefore, non-parametric statistical procedures were employed for group comparisons. The Mann–Whitney *U* test was used to compare the pre-test and post-test scores of the experimental and control groups, whereas the Wilcoxon Signed-Rank Test was used to examine within-group changes over time. Effect sizes (*r*) were calculated for the Mann–Whitney *U* and Wilcoxon Signed-Rank tests and interpreted according to Cohen's (1988) guidelines. Statistical significance was set at *p* < 0.05.

Pearson correlation analysis was conducted to examine the relationships between humor skills and peer relationships. In addition, simple linear regression analysis was performed to investigate the predictive role of humor skills on peer relationships. Because the social interaction, bullying, and victimization dimensions represent distinct conceptual constructs, regression analyses were conducted separately for each sub-dimension rather than using a combined peer relationship score. In these analyses, humor skills were entered as the independent variable, whereas peer relationship dimensions were entered as dependent variables (Büyüköztürk, 2009).

Qualitative data were obtained through semi-structured interviews, field notes, and teacher interviews carried out with the parents of the children in the experimental group (Merriam, 2018). Parent interviews were carried out by two university students, who worked as intern at the school, while teacher interviews were conducted by the researcher. The researcher is also the practitioner of 16 creative drama activities designed within the scope of the study. The researcher became acquainted with the subjects during the research process and had no prior relationship. The interviews were held in a quiet and empty classroom in the school environment, each interview lasted an average of 6 min, and audio recordings were made in line with the participant's consent. It was observed that similar themes were repeated during the interviews. Participant validation was not applied due to the implementation process of the research. The responses given to the questions directed to the children were grouped depending on their similarities, while the data were analyzed in line with the themes and sub-themes (Yildirim and Simşek, 2008). In order to ensure reliability, the data were separately coded by an expert working independently of the research, and then compared with the researcher's coding to identify items of agreement and disagreement. The consensus among the encoders was calculated using the Miles and Huberman's (Equation 1):


$$\text{Reliability}=\frac{\left[\text{Total Number of Agreement}\right]}{\left[\text{Agreement+ Disagreement}\right]}\text{x100}$$

When the percentages of agreement between the researcher and the independent encoder were examined, it was understood that the percentages were 85.71%. While determining the reliability in coding, the notes taken by independent encoders should be coded separately and the resulting notes should be reviewed together. Both the consensus within the encoder and the consensus between the encoders should be above 80% (Miles and Huberman, 2016). In addition, responses to assessment questions and children's behaviors following the activity interventions were noted by the researcher at the end of each day.

## 5 Result

The Mann–Whitney *U* test, one of the non-parametric tests, was applied to the comparison of the pre-test and post-test scores of the children included in the study. Results are presented in Table 2.

**Table 2:** Between-group comparisons of peer relationship and humor scores.

Variables	Group		Pre-test					Post-test				
			*x* ±Sd	*U*	*z*	*p*	*r*	*x* ±Sd	*U*	*z*	*p*	*r*
Bully	Exp.	17	50.35 **±** 8.41	152.00	−0.78	0.435	0.13	39.47 ± 5.21	167.50	−0.32	0.746	0.05
	Cont.	21	50.86 **±** 9.82					31.33 ± 10.02				
Social interaction	Exp.	17	33.12 **±** 5.82	140.00	−1.14	0.256	0.18	61.94 ± 3.78	71.50	−3.15	*p* = 0.002	0.51
	Cont.	21	33.76 **±** 4.18					51.81 ± 11.98				
Victim	Exp.	17	9.94 **±** 1.43	127.00	−1.62	0.104	0.26	5.00 ± 1.17	94.50	−2.55	*p* = 0.011	0.41
	Cont.	21	10.57 **±** 1.40					6.90 ± 2.53				
Understanding humor	Exp.	17	7.12 **±** 1.32	162.00	−0.50	0.616	0.08	14.47 ± 1.18	55.50	−3.95	< 0.001	0.64
	Cont.	21	6.90 **±** 1.26					12.19 ± 1.66				
Preferring humor	Exp.	17	30.88 **±** 5.25	177.50	−0.03	0.976	0.01	43.00 ± 2.85	35.50	−4.23	< 0.001	0.69
	Cont.	21	30.81 **±** 4.42					36.90 ± 4.31				
Producing humor	Exp.	17	11.06 **±** 1.95	316.50	−0.45	0.650	0.07	27.47 ± 3.22	32.00	−4.34	< 0.001	0.70
	Cont.	21	11.38 **±** 2.15					21.38 ± 3.31				
Benefiting form humor	Exp.	17	7.35 **±** 1.93	297.00	−1.03	0.303	0.17	18.47 ± 1.33	45.50	−3.97	< 0.001	0.64
	Cont.	21	6.86 **±** 1.35					13.00 ± 4.22				

M ± SS, arithmetic mean ± standard deviation; *U*, Mann-Whitney *U* statistic; *z*, standardized test statistic; *r*, effect size (*r* = *z*/√*N, N* = total sample). The effect size was interpreted as 0.10 small, 0.30 medium and 0.50 large. ^**^p < 0.01; ^***^p < 0.001.

As shown in Table 2, no statistically significant differences were found between the groups in the pre-test comparisons (all *p* > 0.05; *r* < 0.30). On the other hand, post-test results indicate that there are significant differences with moderate and large effect sizes in favor of the experimental group in all variables except bully dimension (*r* = 0.41–0.70).

The results of the Wilcoxon Signed-Rank Test revealed that there were statistically significant differences between the pre-test and post-test scores of all variables in the experimental group (all *p* < 0.001). A significant decrease was observed in bullying and victimization scores, while a significant increase was seen in the social interaction and humor sub-dimensions. Effect sizes are large (*r* = 0.85–0.89). This finding shows that the applied drama-based intervention program has a strong impact on children's peer relationships and humor skills. Statistically significant changes were found in the pre-test-post-test comparisons in the control group as well (all *p* < 0.001). However, the variations in the control group can be explained by developmental progress due to the natural attendance to the preschool education. As a matter of fact, the effect sizes obtained in the experimental group are more consistent and more directly related to the intervention goals (Table 3).

**Table 3:** Within-group changes from pre-test to post-test.

Group	Scale	*N*	Pretest median (Q1–Q3)	Posttest median (Q1–Q3)	*z*	*p*	*r*
Experimental	Bully	17	52 (45.5–53.0)	30 (25.0–35.5)	−3.623	*p* < 0.001	0.88
Experimental	Social interaction	17	33 (30.5–35.5)	64 (58.0–64.5)	−3.624	*p* < 0.001	0.88
Experimental	Victim	17	10 (9.0–11.0)	4 (4.0–6.0)	−3.671	*p* < 0.001	0.89
Experimental	Understanding humor	17	7 (6.0–8.5)	15 (15.0–15.0)	−3.639	*p* < 0.001	0.88
Experimental	Preferring humor	17	32 (27.0–36.0)	44 (43.0–45.0)	−3.520	*p* < 0.001	0.85
Experimental	Producing humor	17	11 (10.0–12.0)	30 (24.0–30.0)	−3.626	*p* < 0.001	0.88
Experimental	Benefiting from humor	17	7 (6.0–9.0)	20 (16.0–20.0)	−3.632	*p* < 0.001	0.88
Control	Bully	21	53 (49.0–57.0)	27 (25.5–35.5)	−3.287	*p* < 0.001	0.72
Control	Social Interaction	21	35 (32.0–36.5)	53 (45.5–58.5)	−3.772	*p* < 0.001	0.82
Control	Victim	21	11 (10.0–11.0)	7 (4.5–9.0)	−3.555	*p* < 0.001	0.78
Control	Understanding humor	21	7 (6.0–8.0)	12 (12.0–12.5)	−4.033	*p* < 0.001	0.88
Control	Preferring humor	21	32 (27.5–34.5)	37 (34.0–40.0)	−4.020	*p* < 0.001	0.88
Control	Producing humor	21	11 (10.0–13.0)	21 (19.0–22.0)	−4.026	*p* < 0.001	0.88
Control	Benefiting from humor	21	8 (7.0–10.0)	14 (12.5–16.0)	−4.026	*p* < 0.001	0.88

Values are reported as Median (Q1–Q3). The Wilcoxon Signed-Rank Test was applied for the pre-test-post-test variation (two-way). The effect size was calculated with the formula *r* = |*z*| / √*n* (0.10 is small, 0.30 is medium, 0.50 is large effect). Outliers were reviewed using descriptive statistics and extreme value lists, consequently, no significant extreme value patterns that would disrupt the analysis were observed.

The results of the Mann-Whitney *U* test applied on the pre-test-post-test difference scores demonstrated that there were significant differences in some sub-dimensions between the experimental and control groups. Given the social impact dimension, the variation scores of the experimental group were significantly higher than the control group (*U* = 72.50, *p* = 0.002, *r* = 0.51). Similarly, moderate and large effect sizes were obtained in favor of the experimental group in the dimensions of understanding humor (*U* = 74.00, *p* = 0.002, *r* = 0.50), preferring humor (*U* = 85.50, *p* = 0.006, *r* = 0.44), producing humor (*U* = 33.00, *p* < 0.001, *r* = 0.70) and benefiting from humor (*U* = 20.00, *p* < 0.001, *r* = 0.76). On the contrary, the difference between the groups in terms of bullying (*p* = 0.354) and victimization (*p* = 0.228) dimensions is not statistically significant (Table 4). The findings suggest that the intervention led to more pronounced improvements compared to the control group, particularly in the areas of humor production and social interaction.

**Table 4:** Between-group comparisons of change scores.

Scale	Experimental median (IQR)	Control median (IQR)	*U*	*z*	*p*	*r*s
Bully	−19 (10.00)	−23 (15.00)	147.00	−0.927	0.354	0.15
Social interaction	29 (8.50)	21 (18.50)	72.50	−3.116	0.002	0.51
Victim	−5 (1.50)	−4 (5.00)	138.00	−1.206	0.228	0.20
Understanding humor	8 (3.00)	5 (2.00)	74.00	−3.113	0.002	0.50
Preferring humor	11 (10.00)	5 (8.50)	85.50	−2.735	0.006	0.44
Producing humor	16 (5.50)	10 (4.50)	33.00	−4.287	< 0.001	0.70
Benefiting from humor	11 (3.00)	7 (4.00)	20.00	−4.678	< 0.001	0.76

The values are presented as median (IQR). *U*, Mann-Whitney *U* statistic; *z*, standardized test statistic; *r*, effect size (*r* = *z*/√*N*). The effect size was interpreted as 0.10 small, 0.30 medium and 0.50 large.

The results of the Wilcoxon Signed-Rank Test applied on the post-test and follow-up test comparisons revealed that there was no statistically significant difference in any of the sub-dimensions in the experimental group (all *p* > 0.05) (*r* = 0.00–0.40). This finding indicates that the gains obtained after the intervention are maintained during the follow-up period (Table 5).

**Table 5:** Maintenance of intervention effects at follow-up.

Group	Scale	*N*	Post-Test (*x* ±SD)	Follow-up (*x* ±SD)	*z*	*p*	*r*
Experimental	Bully	17	29.47 ± 5.21	30,23 ± 5,03	−1.000	0.317	0.24
Experimental	Social interaction	17	61.94 ± 3.78	61,94 ± 3,78	0.000	1.000	0.00
Experimental	Victim	17	5.00 ± 1.17	5,00 ± 1,17	0.000	1.000	0.00
Experimental	Understanding humor	17	14.47 ± 1.18	14.53 ± 1.07	−1.000	0.317	0.24
Experimental	Preferring humor	17	43.00 ± 2.85	43,41 ± 2,57	−1.000	0.317	0.24
Experimental	Producing humor	17	27.47 ± 3.22	27,94 ± 2,70	−1.633	0.102	0.40
Experimental	Benefiting from humor	17	18.47 ± 1.33	18.53 ± 2.06	−1.342	0.180	0.33

M±SD, arithmetic mean ± standard deviation; z, standardized Wilcoxon test statistic; *r*, effect size (*r* = *z*/√*N*). *p*-values were calculated bi-directionally.

Descriptive statistics and Pearson correlation coefficients for the variables included in the study are presented in Table 6. When evaluating the mean and standard deviation values of the variables examined, it is understood that children's humor skill levels (*M* = 93.11, SD = 12.61), social interaction levels (*M* = 56.34, SD = 10.48), bullying levels (*M* = 30.95, SD = 8.13) and victimization levels (*M* = 6.05, SD = 2.23) demonstrate a certain distribution. When the relationships among the variables are examined, it is found out that there is a moderate, positive and significant relationship between humor skills and social interaction (*r* = 0.404, *p* < 0.05). On the other hand, there was no significant relationship between humor skills and bullying (*r* = −0.111, *p* > 0.05) and victimization (*r* = −0.204, *p* > 0.05). The results of the regression analyses are presented in Table 7.

**Table 6:** Descriptive statistics and correlations among study variables.

Variables	Mean (*M*)	SS	1	2	3	4
Humor (total)	93.11	12.61	—			
Bully	30.95	8.13	−0.111	—		
Social interaction	56.34	10.48	0.404^*^	−0.411^*^	—	
Victimization	6.05	2.23	−0.204	0.076	−0.052	—

^*^p < 0.05.

**Table 7:** Regression analysis predicting peer relationship dimensions from humor skills.

Dependent variable	*B*	SH	β	*t*	*p*	*R* ^2^	*F*
Bully	−0.071	0.107	−0.111	−0.669	0.508	0.012	0.448
Social interaction	0.336	0.127	0.404	2.652	0.012^*^	0.163	7.032
Victim	−0.036	0.029	−0.204	−1.250	0.219	0.042	1.563

*N* = 38. ^*^p < 0.05 (Total humor score was entered as the predictor variable in all regression models).

Upon evaluating the relationships among the sub-dimensions of peer relationships, it is observed that there is a moderate, negative and significant relationship between social interaction and bullying (*r* = −0.411, *p* < 0.05). It was further determined that the relationships between other variables were not statistically significant (*p* > 0.05). The findings obtained as a result of the correlation analysis revealed that humor skills are significantly related to peer relations, especially the social interaction dimension. Although correlation analyzes present information about the direction and level of the relationships between the variables, they are limited in revealing the predictor nature of the relationships in question. Thus, simple linear regression analyses were implemented in order to more comprehensively investigate the specific effects of humor skills on the sub-dimensions of peer relationships. In this context, the predictor role of humor skills on social interaction, bullying and victimization was tested separately.

The results of the regression analysis indicate that humor skills significantly predict the social interaction dimension (β = 0.404, *p* < 0.05). Humor skills account for 16.3% of the variance in social interaction scores. In contrast, it was determined that humor skills do not have a significant predictive effect on the bullying and victimization dimensions. This finding suggests that humor skills may be particularly associated with positive peer interactions.

Implemented quantitative analyses revealed that creative drama activities had positive effects on preschool children's peer relationships and humor skills. The findings show that humor skills significantly predict children's levels of social interaction.

In order to gain a deeper understanding of these findings and to support the behavioral changes observed in children throughout the process with observations from families and teachers, qualitative data were utilized. In this connection, semi-structured interviews were conducted with the families and teachers of the children in the experimental group. Only mothers participated as parents. For the study, children were coded as C1……..C17. Participants were also named with their children's codes.

The data obtained from the interviews were examined through content analysis and the findings were divided into two categories as “social skills” and “emotional skills” for the first question. For the second question, it was divided into the categories of “problem solving” and “self-expression”. For the last question, the categories of “creating humor”, “using gestures and facial expressions” and “understanding humor” were formed.

The quantitative findings of the study revealed that children engaging in drama activities showed significant improvements in peer relationships and humor skills. Parental opinions also endorse this finding. Some parents reported that their children became more willing to communicate with their friends and build up friendships. For example, the mother of child coded (C13) described the change in her child's social skills as follows: “*At the beginning, my child only played with one person. Now they have more friends. Says the names of all of them. My child plays with everyone*.” Similarly, some parents pointed out that their children's social behaviors such as sharing, waiting in line and cooperation increased. One parent described this change in the following words: “*C1 already got along well with his/her older brother normally, but that situation has improved even more here. Level of waiting in line and sharing has increased as well*.” (C1) Some families reported that while children's social skills improve, their emotion regulation skills are also supported. For example, the mother of child C4 stated: “*C4 would get extremely angry when things didn't go his/her way and would yell at everyone at home. C4 has improved a lot now*.” Another child's (C17) mother made these statements: “*C17 used to have difficulty communicating because he/she was shy, but now he/she can communicate a little more comfortably. I am happy to see his/her communication with the friends. C17 has improved quite a bit*.”

Another important finding of the study is that children begin to develop more constructive problem-solving strategies in the face of disagreements with their peers. Parents reported that their children behave more calmly in disagreements and try to solve problems by communicating. For example, the parent of (C2) explained this situation as follows: “*He/she finds a solution by being calmer and thinking than before*.” Similarly, 7's mother stated that her child is now trying to resolve disputes by communicating: “*He/she always started to solve them by talking. “That's how we do it at school,”* he/she says”. There were also responses reported about self-expression and self-defense in face of resolving conflict situations. For example, the mother of C14 said, “*Because C14 has a bit shy characteristics, C14 couldn't fully defend and express oneself on this issue. Now C14 standing up for oneself, while expressing what's bothering him/her.”* C7's mother, on the other hand, stated: “*When someone didn't include C17 in the game, he/she started asking, “Why aren't you playing with me?””*

Findings on humor skills also indicate that drama activities have a positive impact on children's use of humor. According to parent interviews, it also shown that there is an increase in understanding and creating humor, and in using gestures and facial expressions. For example, the parent of (C12) described the change in their child's use of humor as follows: “*C12 is making funny things, saying funny words. He/she makes us laugh*.” Another parent expressed that their child (EC11) has started using humor more in social interactions: “*When something happens and we're feeling down, EC11 tries to make us laugh*.” Additionally, the classroom teacher observed that the children's participation in group activities increased and that they were able to build up collaborative games. Indeed, the teacher explained this situation with the following words: “*The other day, during free-time activity, they were divided into two separate groups. They built up such a harmonious game. The cooperation among them was excellent*.” Similarly, the teacher noted that the children made efforts to communicate more and resolve problems among themselves when in disagreement situations. “*They're trying to resolve things on their own. Some are asking for help*.” Findings on humor skills are also supported by teacher observations. The teacher expressed that the joking behaviors of the children increased in the classroom environment and that they started to express themselves by using gesture-mimics.

The researcher noted that while the children did not utter their feelings or even their names during the first days of the activities, over time they started to oppose injustice and produce effective solutions to their problems, made jokes and laughed at the jokes made since the fifth activity. While they remained silent in the first few days, in the fifth event, when were asked “what would you say if they were Black and no one sold anything to you” C13 replied, “*Are you the only beautiful one? I'm beautiful too*.” In the sixth event, there were short attempts to persuade the Rabbit, such as “*sharing is caring*,” and “*please share*.” C12 angrily said, “*What difference would it make if you gave it to me...Good heavens*!” C13 also said, “*You have plenty of them, nothing would happen if you gave them to us?*” During the process, the children exhibited behavior of defending each other's rights. For example, in the eighth activity, the researcher said that each of them had clothes, which she washed and hung on a line. However, pointing to two volunteer university students, who had participated in the activity that day, she said that she didn't like their clothes and wouldn't hang them up, leaving them wet. In the meantime, C15 said, “*Take those too and paint them whatever color you like*.” Then KC13 said, “*Don't be unfair, you should hang them for drying too*.” Act of humor was also observed during this event. One of the children lined up side by side, Child 10, said, “Look, the teacher is treating them badly, let's run away.” Child 16 then replied, “*We're like laundry, we're hanging on a clothesline, how are we going to run away?*” They also used humor in defending their rights in the thirteenth activity. For the situation of an elderly woman getting angry that they were playing in the park, C3 told them to “*Put on your headphones*,” and also C12 told them to “*Go home and rest*.”

## 6 Discussion

The findings of the study indicate that the change scores for the experimental group were significantly higher than those of the control group in the social interaction dimension—a subdimension of peer relationships—among children participating in creative drama activities (*U* = 72.50, *p* = 0.002, *r* = 0.51).

The findings are consistent with previous studies reporting that creative drama enhances children's social-emotional skills, friendships, cooperation, and social competence (Ceylan and Ömeroglu, 2012; Akalin and Boz, 2024; Karaoglu, 2025; Cam-Türkan, 2026). In addition, Senol and Metin (2021) reported that drama education enhances social interaction and sharing behaviors in preschool children; Korošec and Zorec (2019), meanwhile, reported that creative drama and puppet activities increase social interaction while reducing aggressive behavior.

Peterson et al. (2020) noted that sociodramatic play offers children the opportunity to try out social strategies, interact with their peers, and structure their social relationships. Similarly, Robertson et al. (2020) emphasized that dramatic play supports communication, collaboration, and perspective-taking skills through the rich dialogues, peer collaboration, and continuous social interaction it entails. In a more recent study, Boal-Palheiros and Ilari (2023) reported that drama-based interventions have positive effects on cooperation, self-regulation, and social assertiveness. These findings demonstrate that creative drama can help children develop various social and interpersonal skills that support their social interactions.

In contrast, the difference in intergroup variation on the bullying (*p* = 0.354) and victimization (*p* = 0.228) dimensions is not statistically significant. No significant difference was found between the groups on the bullying dimension; while a significant difference in favor of the experimental group was observed in the final test scores on the victimization dimension, a comparison of the change scores revealed that the intervention had no significant effect (*p* > 0.05). This finding is partially consistent with the findings of Ozen-Uyar et al. (2025). The researchers reported that drama-based interventions improved children's social understanding and social problem-solving skills but did not result in meaningful changes across all dimensions of peer relationships. These results suggest that drama-based interventions may be more effective in fostering positive social processes, while their impact on negative peer behavior may require longer-term interventions or different types of supportive interventions.

The qualitative findings also supported the quantitative results. Parents reported that their children had become more willing to interact with their peers and participate in social activities. For example, one parent noted that their child had previously complained that their peers did not want to play with them, but after the intervention, the child frequently mentioned friends who invited them to play. In the present study, the researcher skipped C8 and moved on to the next child while gathering their opinions during the eleventh activity. At that moment, when the child's facial expression changed, C7, who had been rude to their friends in the previous stages, sadly touched the friend's shoulder and said, “*Didn't the teacher make you tell them?” Then, defending their friend, he/she said, “Excuse me, but, you didn't ask my friend about this.”* In addition, the classroom teacher also said the following words: “*To be honest, some of the children are making progress. They're trying to resolve things on their own. Some are asking for help. Those I'm referring to as asking for help were previously very reluctant and unwilling to do so. But others have taken it a step further. They're trying to find their own way. They're speaking up. They're trying to communicate. They're taking a stand. And if that doesn't work, they come to me for help*.” These observations indicate that the positive changes identified in the quantitative analyses are also reflected in the children's daily social interactions.

The results of the study also revealed moderate to large effect sizes in favor of the experimental group regarding children's humor skills.(Understanding humor [*U* = 74.00, *p* = 0.002, *r* = 0.50], Preferring Humor [*U* = 85.50, *p* = 0.006, *r* = 0.44], Producing Humor [*U* = 33.00, *p* < 0.001, *r* = 0.70], and Benefiting from humor [*U* = 20.00, *p* < 0.001, *r* = 0.76]).

The findings suggest that creative drama may play an important role in the development of children's sense of humor. This can be explained by the fact that, by its very nature, creative drama offers children opportunities to role-play, engage in symbolic thinking, improvise, use their imagination, and create unexpected situations. Sakaryali et al. (2024) note that humor is closely linked to social-emotional development, contributes to the development of interpersonal relationships, and supports children's adaptation to their social environments. Bergen (2019) notes that humor most often emerges within play-based social interactions and that pretend play, imaginary scenarios, and playful communication provide important opportunities for children to understand and generate humor. Similarly, Semrud-Clikeman and Glass (2010) emphasize that the development of humor is closely related to skills such as symbolic play, perspective-taking, social interaction, and recognizing incongruities. Hammershøj (2021), on the other hand, states that play and humor share common characteristics in terms of the processes of creativity, imagination, and the generation of unexpected meanings. In this context, creative drama provides a learning environment where children not only observe humor but also actively create and experience it. It is also noted that humor is closely linked to gaming experiences and serves as an important source of motivation that increases children's participation in activities (Rudenskyi et al., 2024). For this reason, it is believed that the interactions that take place during creative drama activities support children's ability to understand, generate, and use humor in their daily social interactions. The qualitative findings supported the quantitative findings. For example, parent noted that their child has started using humor through gestures and facial expressions: For C7, “*Sometimes when he/she saying something, he/she touches my shoulder, rolling their eyes and says*, “*Mom, you made the food so well.” It's very strange to me. This must be from drama*”. At the start of the activity, none of the children understood humor or made jokes. At the eighth activity, when a child didn't return from the toilet, C2 made funny gestures and changed their voice, saying, “*Maybe he/she fell into the toilet*.” All the children laughed. C16 raised their hands and said, “*No way (that can't be)*.”

The study found that humor skills significantly and positively predict social interaction. This finding demonstrates that humor is not merely a source of entertainment but also an important social skill that supports interpersonal interaction. To use humor effectively, children need to understand the feelings and thoughts of others, interpret social cues, and respond appropriately in social interactions. Indeed, Celume et al. (2020) have shown that drama-based interventions enhance children's theory of mind and cooperative behavior. The ability to understand others' perspectives and to collaborate can be viewed as shared processes that support both the use of humor in a social context and the development of positive peer interactions. Similarly, Paine et al. (2021) note that sharing humor integrates cognitive, social, and emotional processes and is associated with social competence. Researchers also emphasize that humor fosters positive bonding and promotes social harmony in close relationships.

In support of this view, it has been noted that humor is an important component of social competence, can facilitate peer acceptance, and can contribute to the development of friendships (Sletta et al., 1995; Klein and Kuiper, 2006). In addition, it is noted that humor facilitates communication, supports social interactions, and contributes to the development of social closeness among individuals (Chadwick and Platt, 2018). The fact that laughter and humorous interactions during the preschool years mostly occur among peers also supports humor's role in fostering social bonds (Cekaite and Andrén, 2019). Furthermore, it has been reported that humor is closely related to empathy, social cognition, and mentalization processes (Halfpenny and James, 2020; Mazzocconi and Priego-Valverde, 2025) and that early humor skills can predict subsequent social cognitive development (Soy Telli and Hoicka, 2022). From this perspective, it is to be expected that children with well-developed humor skills will communicate more effectively with their peers, participate more actively in social interactions, and develop positive peer relationships.

## 7 Conclusions

This study investigates the effect of creative drama-based intervention on preschool children's peer relationships and humor skills. The results of the analysis reveals that there is no significant difference between the pre-test scores of the experimental and control groups in all variables (all *p* > 0.05; *r* = 0.01–0.26). This indicates that the initial levels of the groups were similar to each other. In the post-test comparisons, significant differences were found out in favor of the experimental group in the social interaction sub-dimension of peer relationships and in all sub-dimensions of humor skills (sense of humor, humor preference, humor creation, and the usefulness of humor) (*p* < 0.05). The calculated effect sizes ranged from moderate to large (*r* = 0.41–0.70), which accordingly indicates that the implemented intervention had a strong effect. On the other hand, no significant difference was found between the groups in the bullying sub-dimension (*p* > 0.05).

Comparison of pre-test-post-test difference scores revealed that the intervention made a supplemental contribution beyond natural development, especially in the dimensions of social interaction and humor. This finding suggests that drama-based interventions have a unique function in terms of strengthening children's peer interactions and supporting humor creation processes. The lack of a significant difference in the bullying and victimization sub-dimensions may be related to the structural characteristics of the preschool classroom environment. Teachers' ability to quickly intervene in peer conflicts and assume a regulatory role in classroom interactions may be reason for having limited variability in these dimensions.

Improvements were observed in some variables of the control group as well. This may be related to children's attendance at regular preschool education, their presence in a structured classroom environment, and their developmental maturation processes. Natural progress is expected over time in social-emotional and humor skills in early childhood. However, when the findings are evaluated together, it can be claimed that the development observed in the experimental group cannot be explained solely by the natural developmental process, and that the implemented drama intervention made a supplemental and significant contribution, especially in terms of social interaction and humor skills. Simple linear regression analyses also put forward that humor skills are related to the positive dimensions of peer relationships. The findings revealed that humor skills positively and significantly predicted social skills; however, they did not have a significant impact on bullying and victimization. The findings of the present study suggest that drama-based interventions may support preschool children's peer relation and humor-related skills.

## 8 Limitations and recommendation

The study has some limitations as well. First of all, the drama activities used in the study were developed by the researcher, and it is recommended to retest them on different practitioners and different samples. Although the sample size meets the minimum requirement specified in the power analysis, the relatively small number of participants should be taken into account when interpreting and generalizing the findings. The fact that the study was conducted with children from families of moderate socioeconomic status in the Sungurlu district of Çorum Province, Turkey, was identified as a limitation. In future studies, it is recommended to conduct similar studies across different socioeconomic levels, in different cultural regions and with larger samples.

Another limitation of the study is that both peer relationships and humor skills were assessed using teacher-administered measures. The use of a single data source may have increased the risk of common method variance and single-source bias. Future studies may benefit from including multiple sources of information, such as parents and teachers, to obtain a more comprehensive assessment of children's behavior.

The questionnaires related to peer relationships and humor skills are based on teacher evaluations, which does not completely eliminate the influence of subjective interpretations in the assessments. Qualitative data were collected only from the experimental group; therefore, no qualitative comparisons could be made between the experimental and control groups.

Also, the interview conditions that emerged during the parent interviews may have limited the duration of some interviews and the level of detail for the data obtained. Since the study was conducted with a limited number of participants, the generalizability of the findings should be carefully evaluated. Furthermore, the participants were grouped in line with their current class structures, thus, full randomization could not be achieved.

In addition, the field notes were recorded by the researcher who conducted the intervention; therefore, the possibility of researcher bias in the interpretation of the observational data cannot be completely ruled out. Due to the nature of the drama-based intervention and the parental informed consent process required in studies conducted with children, the study failed to implement a blinding procedure. Therefore, the findings should be interpreted in the context of the natural education environment. Longitudinal studies investigating the long-term effects of drama activities can be conducted.

## Data Availability

The raw data supporting the conclusions of this article will be made available by the author upon reasonable request.
