# Supplementary material for: The effects of creative drama activities on preschoolers' peer relationships and humor skills and the predictive role of humor
Source: Front Psychol. 2026 Jul 20;17:1892073. doi: 10.3389/fpsyg.2026.1892073 (PMC13431261; doi:10.3389/fpsyg.2026.1892073)
Supplement: Supplementary file 1 [file Data_Sheet_1.docx]

APPENDIX 1

**SUMMARY OF DRAMA SESSİONS**

| **Activity Name** | **Summary** |
| --- | --- |
| **Emotions Park** | **Warm-up:** The children exchanged funny greetings and walked whilst expressing different emotions (happy, sad, angry, scared, surprised, etc.) with their bodies to music. They discussed the situations in which these emotions might arise.  **Role-play:** The children acted out situations such as waiting in a queue, feeling scared, getting angry and apologising in a fictional setting called ‘Emotions Park’. Throughout the activity, their ability to express their own emotions, understand others’ emotions and communicate appropriately was supported. The session concluded with a fun competition in which the children tried to make each other laugh.  **Evaluation:** Discussions were held with the children about how different emotions feel, the effects that others’ negative behaviour has on them, and how they might respond in such situations. |
| **The Lonely Snake** | **Warm-up:** The children formed a circle and expressed their names through different emotions (happy, angry, surprised, scared) and different roles (an elderly person, a baby, a sick person, a funny person).  **Role-play:** The children acted out the roles of animals living in the forest and shared their characteristics. They discussed the feelings experienced by a snake who was excluded by his friends because he smelled bad; the children were encouraged to empathise by taking the snake’s place. The children then expressed their own negative traits in a fun way and took part in an imaginary bath activity with the snake.  **Evaluation:** The children discussed the feelings experienced when facing exclusion, loneliness and not being included in games by their friends; the importance of acceptance, empathy and inclusive behaviour in peer relationships was emphasised. |
| **The Trash That Won’t Go Away** | **Warm-up:** The children took on the roles of rubbish and refuse collectors, moving to the music to act out the recycling process. Throughout the activity, cooperation, active play and fun interactions were encouraged.  **Role-play:** The children took on the roles of various items of rubbish and humorously explained why they had been thrown away. They then acted out a story in which the rubbish, having become friends, did not want to be separated; the children moved together to try to persuade the refuse collector. Throughout this process, cooperation, friendship and problem-solving skills were supported.  **Evaluation:**We discussed the most entertaining characters with the children and explored what they might feel when faced with an unwanted situation, as well as how they could express themselves. |
| **Different But Together** | **Warm-up:** Accompanied by music, the children danced whilst experiencing different physical limitations (such as not being able to see, hear, or use their arms or legs). This helped them to develop awareness and empathy towards differences.  **Role-play:** The children acted out the difficulties a new classmate with a disability might face in daily life. They improvised scenarios—such as hanging up a coat, washing hands and moving around the classroom—to explore how they could help their friend, and worked together to find solutions to these challenges. Throughout the process, the importance of cooperation, empathy and peer support was emphasised.  **Evaluation:** Discussions were held with the children on how to include friends with different abilities in games, and on behaviours related to asking for and offering help. Furthermore, they were encouraged to express situations they were unable to manage in a light-hearted manner, thereby fostering a sense of humour. |
| **Funny Shopping Day** | **Warm-up:** The children acted out various jobs to music, then danced with creative movements based on the theme of spending money.  **Role-play:** The children went shopping using the cards they were given as money. During the activity, improvisations took place when a customer was prevented from shopping due to a physical difference; the children were encouraged to come up with solutions regarding justice, equality and acceptance. The children also tried to get a discount from the shopkeeper using humorous expressions.  **Evaluation:** A discussion took place with the children about the things they wanted to own and what they would like to buy for others. Throughout the activity, the focus was on sharing, respecting differences and the role of humour in fostering positive communication. |
| **The Rabbit Who Won’t Share** | **Warm-up:** The children acted out the roles of different animals living in the forest, following the instructions to prepare for the activity.  **Role-play:** The children acted out the story of a rabbit who wouldn’t share his carrots with anyone. By taking on the roles of different animals, they tried to persuade the rabbit to share, but the rabbit refused. Later, when the unshared carrots went off, the rabbit realised the consequences of its behaviour, apologised to its friends and learnt the importance of sharing. They were expected to use humour to persuade the rabbit. Throughout the process, the focus was on empathy, sharing and friendships.  **Evaluation:** We discussed with the children the feelings experienced when things are not shared, and which items or materials could be shared. |
| **The Polluted Sea** | **Warm-up:** Following a short video introducing marine life, the children acted out creatures such as fish, sea snakes, octopuses and dolphins using body movements.  **Role-play:** The children took on the roles of various marine creatures and improvised a scene depicting the problems faced in a polluted sea. The focus was on the difficulties marine creatures face due to waste and the emotions they experience; the children assessed the effects of environmental pollution on living creatures from different perspectives. A large fish asked the small fish for help, using humour.  **Evaluation::** The children were asked to create a piece of work using waste materials that reflected the role-play. |
| **The Laundry That Won’t Dry** | **Warm-up:** The children took on the role of laundry, bringing to life the clothes being washed in the washing machine. They adapted their movements to the changes in the music, performing fun and creative movements.  **Role-play**: The children took on the role of laundry to be hung out to dry on a line. Through disagreements over whose turn it was to be hung out to dry, the children improvised scenarios focusing on waiting their turn, respecting others’ rights and finding solutions together. Throughout the process, the children were encouraged to listen to different viewpoints, express themselves and reach a compromise with their peers.  **Evaluation:** The children discussed waiting their turn, the protection of rights and the emotions felt in situations of exclusion. |
| **Flower Shop** | **Warm-up:** The children acted out the process of a seed turning into a flower; they prepared for the activity with movements such as growing, swaying in the wind and spreading lovely scents into the surroundings. They also took part in creative movements by acting out bees visiting the flowers.  **Role-play:** The children represented different flowers in a flower shop and explained why they were beautiful. They were encouraged to introduce themselves in a fun and creative way. Subsequently, through improvisations based on the fact that some flowers were not chosen, discussions took place on emotions, respect for differences and appropriate forms of communication.  **Evaluation:** Discussions were held with the children about the qualities they admire in themselves, the feelings they might experience when faced with criticism, and appropriate ways to respond. Furthermore, the most entertaining characters in the activity were evaluated, and humorous storytelling was encouraged. |
| **The Slow Turtle Family** | **Warm-up:** The children acted out different animals living in the forest; they prepared for the activity by moving like animals that were in a hurry, getting tired, or fleeing from danger.  **Role-play:** The children acted out the story of a family of turtles who were always late and, as a result, missed out on important opportunities. The family, who missed school, games and various activities, sought help from the Wise Owl to find solutions to their problems. The aim was for the children to understand the humorous behaviour of the tardy tortoise.  **Evaluation:** A discussion was held with the children about being ready on time, the consequences of being late and what can be done to prevent such situations. They were also encouraged to share their everyday experiences. |
| **The Rejected Slipper** | **Warm-up:** The children took on the role of a pair of shoes and danced with their partners to music; they then portrayed a shoe that had lost its partner, using their bodies to express different emotions.  **Role-play:** The children represented different shoes in a shoe shop and explained why they should be chosen. Improvisations were performed on the emotions experienced by a slipper that was not chosen by customers and was teased by its friends.  **Evaluation**: The children discussed the feelings one might experience when faced with rejection or disapproval, and debated how one might react in such situations. The funniest slipper was chosen. |
| **The Talking Trees** | **Warm-up:** The children acted out the process of a seed turning into a tree; they moved as growing saplings and as trees coming together to form a forest. They prepared for the activity by using their bodies to express the effect of the wind.  **Role-play:** The children introduced themselves by taking on the roles of different trees. They then acted out the emotions trees experience in the face of environmental problems caused by humans; they improvised scenes depicting situations such as damage to their branches, fires being lit and the threat of being cut down. They were asked to persuade people through humour.  **Evaluation:** Discussions were held with the children about protecting nature, environmental responsibility and the emotions felt in the face of harmful behaviour. They also discussed how they could express themselves in situations that caused them distress. |
| **The Angry City** | **Warm-up:** The children walked whilst expressing different emotions with their bodies to music, and then acted out everyday activities using exaggerated and playful movements.  **Role-play:** Through improvisation, the children explored what they might feel and how they might react when faced with negative behaviour. They expressed their feelings through scenarios such as having their balls burst or being shouted at by a stranger, and reflected on appropriate ways to communicate. At the end of the activity, the children came up with funny games and entertaining ideas to help alleviate boredom.  **Evaluation:** A discussion took place with the children about how to support friends who are feeling sad or angry. |
| **The Sad Kites** | **Warm-up:** The children acted out kites of different colours and characteristics; they prepared for the activity with movements such as gliding through the sky, speeding up and dancing.  **Role-play:** The children introduced themselves as kites with different characteristics. They then improvised scenes based on the experiences and feelings of a kite that was not wanted to fly with the others because it was different. The children developed suggestions to help the excluded kite feel better and build positive relationships with its peers.  **Evaluation:** The children discussed topics such as exclusion, not being included in games and choosing friends. The funniest kite was discussed. |
| **The Living Toys** | **Warm-up:** The children brought the toys in a child’s bedroom to life. Movement exercises were carried out based on the idea that the toys come to life and play together whilst the child is asleep, and freeze when the child wakes up.  **Role-play:** The children improvised a story about a puppet who was sad because nobody was playing with him. They discussed the emotions the puppet was experiencing and came up with words and behaviours that might make him feel better.  **Reflection:** The children discussed the feelings one might experience when being excluded or not included in a game. |
| **The Clock Shop** | **Warm-up:** The children examined different images of watches and discussed who might use them. They then acted out working and broken watches using body movements, accompanied by music.  **Role-play:** The children took on the roles of watches with different features, introduced themselves as if in a watch shop, and explained why they should be chosen.  **Evaluation:** The children discussed their favourite watches, the reasons for their choices, and which watch they would like to be. The funniest watch was discussed. . |

**APPENDIX 2**

**Parent Interview Questions**

1. What kinds of changes have you observed in the way your child interacts with peers and initiates, maintains, and develops friendships (such as sharing, cooperating, waiting for their turn, and making friends)?
2. Have you noticed any changes in your child’s problem-solving strategies, behavior toward peers, or reactions to how others treat them when they experience conflicts with their peers? Could you explain?
3. Have you observed any development or changes in your child’s ability to use humor in daily life (such as making jokes, understanding jokes, responding appropriately, or using gestures, facial expressions, or tone of voice to make others laugh)? Can you provide an example?

**Teacher Interview Questions**

1. What kinds of changes have you observed in the way children communicate with one another and participate in group activities (such as sharing, cooperating, waiting their turn, and adapting)?
2. Have there been any changes in the children’s problem-solving strategies when faced with conflicts with their peers, in their behavior toward their friends, or in their reactions to how others treat them? Could you explain?

Have you noticed any changes or improvements in the children’s ability to use humor in the classroom setting (such as making jokes, understanding jokes, responding appropriately, or using gestures, facial expressions, or tone of voice to make others laugh)?
